# Syntactic processing as a marker for cognitive impairment in amyotrophic lateral sclerosis

**DOI:** 10.3109/21678421.2015.1071397

**Published:** 2015-08-27

**Authors:** Stella Tsermentseli, P. Nigel Leigh, Lorna J. Taylor, Aleksandar Radunovic, Marco Catani, Laura H. Goldstein

**Affiliations:** ^a^ Faculty of Education and Health, Department of Psychology, Social Work and Counselling, University of Greenwich, London, UK; ^b^ Brighton and Sussex Medical School, Trafford Centre for Biomedical Research, University of Sussex, Falmer, UK; ^c^ The Michael Rutter Centre, South London and Maudsley NHS Foundation Trust, London, UK; ^d^ Barts Health Motor Neurone Disease Centre, Barts Health NHS Trust, London, UK; ^e^ King's College London, Institute of Psychiatry, Psychology and Neuroscience, Natbrainlab, Department of Forensic and Neurodevelopmental Sciences, London, UK; ^f^ King’s College London, Institute of Psychiatry, Psychology and Neuroscience, Department of Psychology, London, UK

**Keywords:** Cognition, language, speech, amyotrophic lateral sclerosis

## Abstract

Despite recent interest in cognitive changes in patients with amyotrophic lateral sclerosis (ALS), investigations of language function looking at the level of word, sentence and discourse processing are relatively scarce. Data were obtained from 26 patients with sporadic ALS and 26 healthy controls matched for age, education, gender, anxiety, depression and executive function performance. Standardized language tasks included confrontation naming, semantic access, and syntactic comprehension. Quantitative production analysis (QPA) was used to analyse connected speech samples of the Cookie Theft picture description task. Results showed that the ALS patients were impaired on standardized measures of grammatical comprehension and action/verb semantics. At the level of discourse, ALS patients were impaired on measures of syntactic complexity and fluency; however, the latter could be better explained by disease related factors. Discriminant analysis revealed that syntactic measures differentiated ALS patients from controls. In conclusion, patients with ALS exhibit deficits in receptive and expressive language on tasks of comprehension and connected speech production, respectively. Our findings suggest that syntactic processing deficits seem to be the predominant feature of language impairment in ALS and that these deficits can be detected by relatively simple language tests.

## Introduction

There is currently great interest in characterizing the cognitive profiles of patients with amyotrophic lateral sclerosis (ALS) who do not meet criteria for frontotemporal lobar degeneration (FTLD) (1). Considering that degeneration in ALS affects primarily posterior frontal regions, this line of research has mainly focused on the presence of executive functions (e.g. (2–4)). Nevertheless, in recent years research has shown that cognitive deficits go beyond an executive dysfunction syndrome and reveal that language is also affected (5–7). A variety of structured language tests have been used to detect linguistic impairment in ALS and the most common changes reported include confrontation naming, syntactic comprehension and verb processing (8–11). Although there is some clinical utility in using standardized tests, this approach oversimplifies the complexity of the linguistic data as these tests do not evaluate functional language skills such as discourse.

Recent work has documented deficits in the ability of ALS patients to organize the local connectedness and maintenance of the theme during narrative discourse (12). However, the specific nature of these deficits in ALS is not yet clear. This is critical because spontaneous connected speech is an essential part of everyday communication. Features of connected speech in ALS have only been investigated by one recent study that reported a deficit in grammatical expression (13). The present study aimed to build on these recent findings (13) and improve the characterization of connected speech production and other language functions in ALS. A secondary aim was to examine the potential of formal standardized language testing and spontaneous speech analysis in the identification of a specific linguistic marker for ALS.

## Materials and methods

### Participants

Twenty-six patients with sporadic ALS and 26 healthy controls participated in the study. ALS patients were mainly recruited from The King's Motor Neurone Disease (MND) Care and Research Centre, London, UK. Additional patients were recruited from Barts Health Motor Neurone Disease Centre in London and from the Motor Neurone Disease Association (MNDA) research register. Healthy control participants were recruited through a volunteer database and the local community.

Inclusion criteria for all participants were age < 75 years, English as a first language, right handedness, no history of cerebrovascular disease, hypertension, diabetes or head injury, not taking any psychoactive medication and IQ > 70. We included patients with probable or definite ALS (14) within 24 months after diagnosis. Patients with bulbar symptoms (ALSFRS-R (15) score on bulbar items 1–3, < 9), respiratory insufficiency (FVC < 70% predicted), and insufficiently intelligible speech were excluded. Additional patient exclusion criteria included a clinical diagnosis of Frontotemporal Dementia (FTD), Primary Progressive Aphasia (either progressive non-fluent aphasia (PNFA) or semantic dementia (SD)) – according to FTLD diagnostic criteria (16). Control participants were matched as closely as possible to the ALS group for age, gender and education.

### Measures

Patients’ functional abilities were assessed using the revised Amyotrophic Sclerosis Functional Rating Scale (ALSFRS-R) (15). The Wechsler Abbreviated Scale of Intelligence (WASI) (17) and Wechsler Test of Adult Reading (WTAR) (18) were used to assess current and premorbid IQ, respectively. Symptoms of anxiety and depression were measured using the Hospital Anxiety and Depression Scale (HADS) (19). The Family Form of the Frontal System Behaviour Scale (FrSBe) was used to assess behavioural changes (20), characterized as scores on subscales of Apathy, Executive Dysfunction and Disinhibition.

The neuropsychological data for the present study included a broad battery of tests assessing executive function, visuoperceptual ability, memory and language. To determine differences in cognitive functioning outside the language domain between patients with ALS and healthy controls, composite scores were calculated for the Executive, Visuoperceptual and Memory domains (Table I). This was undertaken with the aim of reducing the likelihood of making type 1 errors through multiple comparisons using each individual neuropsychological test score, following the methods described by Taylor et al. (6).

**Table I.** Test indices included in cognitive composites.

Domain	Measures contributing to the composite (scores reflected where necessary so that higher composite scores indicated greater impairment).
Executive functioning	Phonemic Verbal Fluency Index (VFi) (3): Words beginning with ‘S’ produced in 5 min and 4-letter words beginning with ‘C’, produced in 4 min adjusted for motor disability. Semantic VFi (3): ‘Animal’ and ‘Food’ words, adjusted for motor disability produced in 2 min, respectively. Hayling Sentence Completion Test (27): Latency score (i.e. the difference between the time taken to complete both sections of the test, ‘Unconnected Sentences’ total time’ – ‘Sensible Completion’ total time). Test of Everyday Attention (28): Telephone search dual-task decrement score. Computerized Wisconsin Card Sorting Test (WSCT) (29): Categories achieved, trials to first category Composite's Cronbach's alpha = 0.664
Memory	California Verbal Learning Test (CVLT) (29): free recall, short-delay free recall, long-delay free recall, long-delay cued recall scores Composite's Cronbach's alpha = 0.771
Visuoperceptual Ability	Visual Object and Space Perception Battery (VOSP) (30): total number of errors on object decision and position discrimination subtests. Judgment of Line Orientation (JLO) (31): total numbers of errors Composite's Cronbach's alpha = 0.617

Formal standardized language testing included measurements of confrontation naming, semantic access, and single word and syntactic comprehension. Confrontation naming was measured with the Graded Naming Test (21). Semantic access of nouns/objects and verbs/action was measured by the Pyramids & Palm Trees (22) and the Kissing & Dancing Tests (22), respectively. Syntactic and single word comprehension was assessed with the Test of Reception of Grammar (24), the modified Token Test (25) and the British Picture Vocabulary Scale-II (26).

The connected speech sample was taken from the Cookie Theft picture description component of the Boston Diagnostic Aphasia Examination (33). Quantitative production analysis (QPA) (34) was used to analyse the transcribed language samples following the approach used by Wilson et al. (35). Transcripts were coded by two graduates trained in analysis of recorded discourse, blind to participants’ status. Coding showed high inter-rater reliability (correlations ranged from .85 to .93) with the exception of phonological distortions, paraphasias and semantic errors. The identification of distortions and semantic errors has been found to be the most problematic aspect of coding (35). Distortions were defined as articulatory speech errors that did not involve frank phonemic substitutions. For patients with dysarthria, every word could potentially be classified as a distortion, so for these patients only words that were distorted above a patient's most accurate speech were coded as distortions. Semantic errors were recorded when participants produced sentences that were syntactically well-formed, but were either nonsensical or were semantically inappropriate for the context. The most common type of semantic errors involved substitutions of semantically related items. Discrepancies in coding were resolved following advice from a speech and language therapist.

The linguistic profile of connected speech production assessed measures under four main categories: 1) speech production (numbers of words, duration of narratives, speech rate, distortions and phonological paraphasias); 2) disruptions to fluency (false starts, filled pauses, repaired sequences and incomplete sentences); 3) lexical content (proportional frequencies of closed class words, pronouns and verbs); and 4) syntactic structure and complexity (mean length of utterances, proportional frequencies of words in sentences, number of embeddings, and semantic errors).

Ethics approval for the study was obtained from The Joint South London and Maudsley and The Institute of Psychiatry NHS Research Ethics Committee (07/H0807/85). Appropriate institutional approvals were obtained to permit recruitment from additional centres. Informed written consent was obtained from all participants, consistent with the Declaration of Helsinki.

### Statistical analysis

The data were analysed using Statistical Package for the Social Sciences (SPSS) Version 21. Variables were checked for normality and homogeneity assumptions of parametric tests. Between-group comparisons were undertaken using *t*-tests or multivariate analysis of covariance (MANCOVA). Categorical data were analysed using χ^2^ tests. Pearson's correlations and discriminant function analyses were used to examine the relationships between language scores and clinical measures. All tests were two-tailed and statistical significance was set at *p* < 0.05.

## Results

### Background characteristics

Demographic and clinical characteristics of participants are summarized in Table II. The mean ALSFRS-R score at the time of neuropsychological assessment was 40.38 (SD, 4.16). Eighty-five percent of patients had limb onset and 15% had bulbar-onset disease, with an average delay from symptom onset to diagnosis of approximately 10 months (M = 9.26, SD = 7.8). Seventy-six percent of ALS patients were taking riluzole at the time of assessment. There were no significant differences between the groups in terms of age, education, gender ratio, current IQ or estimated premorbid IQ, current FrSBe subscale scores as rated by informants, or HADS anxiety and depression scores. The groups also did not differ in terms of their executive function, memory or visuoperceptual performance as measured by composite score domains.

**Table II.** Demographic and clinical characteristics of participants and composite scores.

	Mean (SD)			
	ALS	Controls	*t* (df)/ χ^2^ (df)	*p*
Gender, *n* (%) Male	23 (88%)	20 (74%)	1.229 (1)	0.268
Age (years)	52.98 (12.13)	48.06 (8.70)	−1.678 (50)	0.100
Education (years)	13.43 (3.82)	14.80 (4.46)	1.197 (50)	0.237
HADS Depression	2.04 (2.34) (Median = 1)	1.88 (2.25) (Median = 1)	−2.42 (50)	0.810
HADS Anxiety	2.73 (2.67) (Median = 2)	3.84 (3.30) (Median = 2)	1.337 (50)	0.187
Current IQ (WASI)	114.18 (14.50)	118.04 (11.28)	1.035 (47)	0.306
Premorbid IQ (WTAR)	111.42 (6.79)	114.84 (7.17)	1.767 (50)	0.083
FrSBe apathy*	48.05 (9.88)	46.13 (12.54)	−0.567 (43)	0.574
FrSBe disinhibition*	50.59 (11.61)	50.91 (11.61)	0.095 (43)	0.925
FrSBe executive dysfunction*	49.23 (9.99)	46.82 (10.20)	−0.791 (43)	0.434
Executive function composite	−0.47 (2.5)	0.01 (1.05)	0.928 (50)	0.358
Memory composite	−0.34 (0.83)	0.01 (0.85)	1.556 (50)	0.126
Visuoperceptual composite	−0.29 (1.68)	0.01 (0.70)	0.865 (50)	0.391

*T score HADS: Hospital Anxiety and Depression Scale; WASI: Wechsler Abbreviated Scale of Intelligence; WTAR: Wechsler Test of Adult Reading; FrSBe: Frontal Systems Behaviour Scale.

The specificity of interpretations drawn from this study may be restricted by the use of cognitive composite scores derived from varied assessment measures. Although this method avoids type 1 errors and allows a more global interpretation based on overall rates of impairment in executive and other domains, further details of scores on individual measures (Table III) provide additional information concerning the profiles of cognitive impairment. Individual measures of executive, memory and visuoperceptual functioning did not differ between ALS patients and controls, with the exception of TEA dual decrement *(p = .*049), the WCST categories achieved *(p = .*052) and CVLT short- delay recall *(p = .*058) which showed marginal differences. These data were not used further in this study. However, after individual inspection of the data, four patients with ALS exhibited impaired phonemic verbal fluency (VFi). These patients scored at least 2 SDs below the mean score of healthy controls on two tests of phonemic verbal fluency.

**Table III.** Means and standard deviations of individual measures of executive, memory and visuoperceptual functioning in ALS and controls.

Test	Raw score, Mean (SD) Controls		Raw score, Mean (SD) ALS		F*	*p*
Executive function						
Category Verbal fluency Index-Animals	1.95	1.02	2.41	1.38	1.057	.311
Category Verbal fluency Index-Food	1.89	1.30	2.05	1.55	.035	.854
S Words Verbal fluency Index	3.25	2.11	4.14	2.94	1.031	.317
4-Letter C Word Verbal Fluency index	9.67	4.86	13.40	8.33	2.986	.093
Hayling Latency	8.05	11.83	14.31	9.40	3.043	.090
TEA dual decrement	1.19	.67	2.17	2.51	4.249	.049
WCST Categories achieved	6.00	.000	5.13	1.89	4.476	.052
WCST trials to first category	19.18	14.84	17.06	9.48	.276	.602
Memory						
CVLT free recall	51.19	9.94	50.38	8.84	.004	.950
CVLT short-delay free recall	.69	1.27	−.08	1.05	4.524	.058
CVLT long-delay free recall	.23	.80	.25	.87	.138	.712
CVLT long-delay cued recall	.10	.86	−.08	1.04	.061	.807
Visuospatial ability						
JLO	27.69	2.51	26.50	4.46	.912	.344
VOSP object decision	18.15	1.29	18.79	1.25	2.507	.120
VOSP position discrimination	19.65	.89	18.71	2.99	2.265	.139

TEA: Test of Everyday Attention; WCST: Computerized Wisconsin Card Sorting Test; CVLT: California Verbal Learning Test; JLO: Judgment of Line Orientation*;* VOSP: Visual Object and Space Perception Battery.

*ANCOVA adjusted for premorbid IQ.

### Connected speech measures

The performance on measures of connected speech production of ALS patients and healthy controls was compared using multivariate analyses of covariance (MANCOVAs). Although the two groups did not differ in terms of premorbid IQ, WTAR-predicted IQ correlated with some language measures and was therefore entered as a covariate. MANCOVA revealed that the patients’ connected speech performance was significantly more impaired than that of healthy controls, λ = 0.402, F(17,30) = 2.631, *p* < 0.01. Post hoc (Bonferroni-corrected) analyses of the univariate contrasts showed that ALS patients produced a significantly lower mean number of words *(p = .*007), lower mean duration of narrative *(p* = .018), a higher mean number of distortions *(p = .*018), and more incomplete sentences *(p = .*009). Patients also produced a lower mean length of utterances *(p = .*004) and more semantic errors *(p = .*014).

To evaluate the contribution of a bulbar motor disorder, we identified mild dysarthria in three patients (ALSFRS-R (15) score on speech item < 4). To minimize the possibility that language performance was influenced by dysarthria or verbal fluency, following the approach of earlier work (13) we separately analysed performance in the subset of 23 patients who did not have dysarthria and the 22 patients who did not have an impaired VFi. The main effect remained significant in the subset of patients without dysarthria (λ = 0.229, F(17,26) = 1.665, *p < *0.01) and in the subset of patients without impaired VFi (λ = 1.506, F(17, 30) = 2.658, *p = *.036). Univariate contrasts showed that non-dysarthric ALS patients produced more distortions *(p = .*038), more incomplete sentences *(p = .*040), lower mean length of utterances *(p = .*004), and more semantic errors *(p = .*029) than controls. ALS patients without impaired VFi also produced more distortions *(p = .*037), more incomplete sentences *(p = .*049), lower mean length of utterances *(p = .*004), and more semantic errors *(p = .*029). Measures of connected speech performance in ALS and controls are summarized in Table IV.

**Table IV.** Mean (SD) measures of connected speech production in all patients with ALS, patients with ALS without verbal-fluency impairment (VFi), non-dysarthric patients with ALS, and controls.

	Controls (*n = *26)	All ALS (*n = *26)	ALS without dysarthria (*n = *23)	ALS without verbal fluency impairment (*n = *22)
Speech rate and speech errors				
Total numbers of words	179.60 (76.10)	123.50** (58.20)	148.25 (61.53)	135.80 (45.88)
Total duration of narrative (in seconds)	80.48 (36.04)	60.62 * (22.65)	73.50 (23.03)	68.60 (20.99)
Speech rate (words per minute)	139.53 (28.16)	120.63 (33.08)	119.18 (33.92)	120.50 (35.12)
Distortions (per hundred words)	.56 (.98)	1.48 * (1.72)	1.36 * (1.75)	1.44* (1.82)
Phonological paraphasias (per hundred words)	.09 (.80)	.25 (.87)	.30 (.95)	.28 (.96)
Disruptions to fluency				
False starts (per hundred words)	.04 (.18)	.06 (.32)	.07 (.35)	.07 (.35)
Filled pauses (per hundred words)	3.75 (2.33)	4.34 (2.87)	4.50 (2.66)	4.36 (2.95)
Repaired sequences (per hundred words)	.90 (.26)	.90 (.94)	.92 (.94)	.89 (.94)
Incomplete sentences (per hundred words)	.09 (.57)	.45** (.57)	.36 * (.50)	.41* (.59)
Lexical content				
Closed class words (proportion)	.14 (.02)	.14 (.06)	.14 (.05)	.14 (.073)
Pronouns (proportions)	.34 (.09)	.32 (.06)	.47 (.06)	.31 (.06)
Verbs (proportion)	.48 (.06)	.48 (.08)	.45 (.08)	.48 (.08)
Syntactic structure and complexity				
Mean length of utterance	18.75 (4.09)	14.28** (4.79)	13.61** (4.48)	13.98 **(4.91)
Words in sentences (proportion)	.11 (.16)	.10 (.09)	.09 (.03)	.11 (.09)
Syntactic errors (per hundred words)	1.11 (1.30)	.86 (.88)	.95 (.92)	.83 (.92)
Embeddings (per hundred words)	.10 (.26)	.09 (.21)	.10 (.23)	.06 (.20)
Semantic errors (per hundred words)	.009 (.05)	.30 * (.56)	.36 * (.60)	.32* (.60)

ALS differs from controls: *p, 0.01; ** *p*, 0.05.

### Formal standardized language testing

A MANCOVA (adjusted for premorbid IQ) showed that ALS patients’ performance on formal standardized language tests was more impaired than that of healthy controls overall (λ = 0.605, F(7,38) = 3.539, *p < *0.01. Univariate contrasts showed that, compared to controls, ALS patients made more errors (*p = *.019), completed fewer blocks (*p = *.020) and scored lower overall (*p = *.022) on the TROG. They also performed worse on the modified Token Test (*p* <.001) and the KDT Test (*p = *.020). These results were also observed in the subset of patients without impairment on VFi (λ = 0.789, F(10,36) = 2.841, *p* < 0.001) and the non-dysarthric patients (λ = 0.475, F(10, 33) = 3.645, *p = *0.002). Means and SDs of structured language tests in ALS and controls are summarized in Table V.

**Table V.** Means (SD) of structured measures of language functioning in all patients with ALS, patients with ALS without verbal fluency impairment (VFi), non-dysarthric patients with ALS, and controls.

	Controls (*n = *26) M (SD)		All ALS (*n = *26) M (SD)		ALS without dysarthria (*n = *23) M (SD)		ALS without verbal fluency impairment (*n = *22) M (SD)	
MTT scores	14.88	(0.43)	13.44**	(1.66)	13.15**	(1.72)	13.26**	(1.85)
GNT scores	23.76	(1.92)	22.42	(4.40)	22.85	(4.72)	22.35	(4.33)
BPVS	151.64	(23.13)	147.79	(17.59)	147.25	(18.81)	146.20	(18.65)
TROG errors	.23	(.71)	1.11*	(1.60)	1.35*	(1.72)	1.40*	(1.72)
TROG blocks completed	19.80	(.63)	18.96*	(1.58)	18.75*	(1.71)	18.70*	(1.71)
TROG standard score	108.07	(2.99)	104.00*	(7.48)	103.00*	(8.05)	102.75*	(8.09)
PPT	51.53	(.64)	50.92	(1.09)	50.95	(.99)	50.90	(1.20)
KDT	51.73	(.66)	50.07*	(1.57)	50.15*	(1.69)	50.05**	(1.70)

MTT: modified Token Test; GNT: Graded Naming Test; BPVS: British Picture Vocabulary Scale; TROG: Test for the Reception of Grammar; PPT: Pyramids and Palm Trees Test; KDT: Kissing and Dancing Test.

ALS differs from controls: *p, 0.01; ** *p*, 0.05.

### Relationship of language deficits with other measures

The relationship between deficits on the language measures with cognitive composites, apathy scores and other measures of motor functioning was investigated using a series of correlations in the patient group only. The group of all patients with ALS exhibited a correlation between duration of narrative and motor speech functioning as measured by ALSFRS-dysarthria score *(r* = .461, *p = *.018). This relationship did not survive when the dysarthric and the VFi-impaired patients were excluded. No other significant correlations were identified.

### Discriminant analysis

A stepwise discriminant function analysis was performed to determine whether specific language measures could be used together to predict group membership (i.e. ALS or control). The measures that were included in the analysis were distortions, incomplete sentences, mean length of utterances, semantic errors, TROG standard score, the modified Token Test score and KDT score. Our results showed that mean length of utterance was entered first, followed by incomplete sentences and then the modified Token Test, with no other measures contributing significantly to discriminability. Ninety-six percent of healthy controls and 81.8% of all ALS patients were correctly classified (Wilks’ Lambda = .537, χ^2^ = 28.28, *p* < 0.001). These data suggest that the three language measures provide a sensitivity of 81.8% and a specificity of 96%. When patients with dysarthria and executive (i.e. VFi) impairment were removed, the same model classified 96% of healthy controls and 85% of ALS patients accurately (Wilk's lambda = .436, χ^2^ = 34.489, *p* < 0.001).

## Discussion

The objective of this study was to clarify whether ALS patients without FTLD presented specific changes in connected speech production evident in a description of the Cookie Theft Picture, as well as other linguistic deficits as measured by a variety of other formal standardized language tests. The findings of the present study are consistent with literature indicating expressive and receptive language disturbances in ALS (6,8–13). We found that patients with ALS showed deficits in speech production fluency and in semantic and syntactic comprehension. Consistent with recent work (13) we also showed that syntactic and action knowledge deficits are independent of motor speech impairments and that syntactic features can be informative in distinguishing between ALS and healthy control groups. In previous studies (6,9,12,13) ALS patients had significantly poorer executive performance than controls, while in ours they performed within the normal range. This difference across research samples might reflect the large variability within the ALS population, and/or our stringent exclusion criteria. Research on less cognitively impaired patients is crucial to isolate the relationship between ALS and language impairment. Thus, on the assumption that our sample was on average less cognitively impaired than previous samples, the current results suggest that syntactic and semantic processing can deteriorate in ALS independently and/or earlier than other cognitive functions.

On the connected speech measures, patients with ALS were impaired on dimensions that contribute to fluency; they produced a lower number of words, a shorter duration of narrative and made more distortions. These deficits did not survive when the dysarthric ALS patients were excluded and were probably the consequence of motor speech deficits. Lexical content was not impaired in any of the subgroups of patients; however, many patients produced utterances that were not complete sentences and showed a reduced length of utterances and more semantic errors. These selective deficits in the absence of executive function difficulties suggest a syntactic complexity impairment, consistent with findings recently reported in the literature (13,36). Interestingly, these linguistic dysfunctions parallel the connected speech features of patients with PNFA reported by Wilson et al. (35).

Performance on structured standardized language tests confirmed that ALS patients showed impairment in syntactic comprehension and semantic/action knowledge, in agreement with previous studies (3,10,35,37), but were relatively unimpaired on tests of single word naming and comprehension of objects. Previous studies (6,9) reported confrontation naming deficits in ALS but these were mild in clinical terms. Our findings again confirm the proposition that ALS patients may have specific impairments of the syntactic and action knowledge systems and also suggest that most of these deficits are not related to executive dysfunction. The present findings relating to sentence production are in agreement with others (13) but are set in the context of a more comprehensive assessment of language function, highlighting the sensitivity of these measures in detecting abnormalities. Difficulties in the mechanics of language are a common characteristic in FTD-ALS with bulbar onset as well as PNFA. Our findings are consistent with previous research that has highlighted both overlap and independence of FTD and aphasia (6,38) and extend the finding of syntactic impairment in ALS patients with limb onset.

It is well known that Broca's area is involved in the processing of sentence structure (39,40). Broca's area is adjacent to motor areas such as the lower precentral gyrus. Yoshizawa et al. (36) found reduced activation in Broca's area in ALS patients with syntactic comprehension impairments. They proposed that in ALS, neurodegeneration first progresses from the medulla oblongata and pons to the prefrontal gyrus and Broca's area. When neurodegeneration is limited to Broca's area, syntactic comprehension impairments without executive dysfunction might occur. This pattern of degeneration could explain why executive and language dysfunction are strongly associated in ALS, and also why executive dysfunction cannot always account for the language impairment observed. Further imaging studies are needed to determine the mechanisms by which neurodegeneration progresses in ALS and how these affect non-motor systems.

The results of our discriminant analysis confirmed that three measures of syntax may differentiate between ALS and healthy controls. Two connected speech measures (i.e. mean length of utterance and incomplete sentences) were the strongest discriminating factors followed by spoken sentence comprehension. From a clinical point of view, progressively deteriorating bulbar symptoms make it difficult to evaluate language and especially speech functions in ALS. In the past, speech related problems in ALS may have been relatively overlooked in linguistic research because evaluation of these features in ALS patients is difficult. In the present study, features of syntactic processing accurately differentiated ALS patients from healthy controls without being masked by dysarthria. Our findings suggest that syntactic processing deficits may be evident at the early stages of the disease and that these deficits can be detected by relatively simple language tests.

Most importantly, the recent interest in the cognitive changes in non-demented ALS has led to the publication of consensus criteria for optimum assessment of cognitive profile (41). These criteria recommend that non-demented patients with ALS be considered as cognitively impaired based solely on measures of executive functioning. Our findings are in agreement with Taylor et al. (6) and suggest that language dysfunction is an important aspect of the cognitive profiles of non-demented ALS patients that cannot always be explained by executive dysfunction. Failure to revise the current consensus criteria could lead to underestimates of the prevalence rates of cognitive impairment and the development of less viable diagnostic, prognostic and monitoring markers.

The results of this study must be interpreted with caution due to the following limitations: 1) the small sample size; 2) the young average age of both groups; 3) the predominantly male sample; 4) the relatively mild motor impairment of the ALS patients; 5) the small number of bulbar-onset patients; and 6) the high intellectual ability of our participants. The pattern of language impairment reported in the present study relates only to relatively unimpaired sporadic ALS patients and therefore the current study cannot comment on the general nature of language impairment in the wider ALS population. Finally, even though a wide range of executive function tests was incorporated, we cannot exclude the possibility that language performance could have been influenced by executive sub-functions (e.g. organization and sequencing) that have not been assessed in the present study.

Despite these limitations, the study confirms that independent linguistic deficits in ALS patients are present and these may be specific rather than global. It appears that the syntactic system is disrupted, while lexical access is relatively spared. This study has also provided the most comprehensive description of connected speech production in ALS to date. We showed that a detailed quantification of connected speech is necessary to characterize the linguistic changes of verbal expression in patients with ALS. The present study also proposes that the linguistic features derived from a simple picture description task can be clinically informative. As we previously highlighted (6), there is a need to reconsider the current consensus criteria (41) for the identification of cognitive impairment in ALS, in order to include language impairments as well as executive dysfunction.
